# Field validation of a magneto-optical detection device (Gazelle) for portable point-of-care *Plasmodium vivax* diagnosis

**DOI:** 10.1371/journal.pone.0253232

**Published:** 2021-06-22

**Authors:** Hugo O. Valdivia, Priyaleela Thota, Greys Braga, Leonila Ricopa, Keare Barazorda, Carola Salas, Danett K. Bishop, Christie A. Joya

**Affiliations:** 1 Department of Parasitology, U.S. Naval Medical Research Unit 6 (NAMRU-6), Lima, Peru; 2 Hemex Health, Inc., Portland, OR, United States of America; 3 NGO PRISMA, Lima, Peru; 4 Vysnova, Lima, Peru; Instituto Rene Rachou, BRAZIL

## Abstract

A major challenge for malaria is the lack of tools for accurate and timely diagnosis in the field which are critical for case management and surveillance. Microscopy along with rapid diagnostic tests are the current mainstay for malaria diagnosis in most endemic regions. However, these methods present several limitations. This study assessed the accuracy of Gazelle, a novel rapid malaria diagnostic device, from samples collected from the Peruvian Amazon between 2019 and 2020. Diagnostic accuracy was compared against microscopy and two rapid diagnostic tests (SD Bioline and BinaxNOW) using 18ssr nested-PCR as reference test. In addition, a real-time PCR assay (PET-PCR) was used for parasite quantification. Out of 217 febrile patients enrolled and tested, 180 specimens (85 *P*. *vivax* and 95 negatives) were included in the final analysis. Using nested-PCR as the gold standard, the sensitivity and specificity of Gazelle was 88.2% and 97.9%, respectively. Using a cutoff of 200 parasites/μl, Gazelle’s sensitivity for samples with more than 200 p/uL was 98.67% (95%CI: 92.79% to 99.97%) whereas the sensitivity for samples lower than 200 p/uL (n = 10) was 12.5% (95%CI: 0.32% to 52.65%). Gazelle’s sensitivity and specificity were statistically similar to microscopy (sensitivity = 91.8, specificity = 100%, p = 0.983) and higher than both SD Bioline (sensitivity = 82.4, specificity = 100%, p = 0.016) and BinaxNOW (sensitivity = 71.8%, specificity = 97.9%, p = 0.002). The diagnostic accuracy of Gazelle for malaria detection in *P*. *vivax* infections was comparable to light microscopy and superior to both RDTs even in the presence of low parasitemia infections. The performance of Gazelle makes it a valuable tool for malaria diagnosis and active case detection that can be utilized in different malaria-endemic regions.

## Introduction

Malaria is an important public health threat that led to more than 228 million cases and 405 000 deaths in 2018 [1]. The disease is caused by five different *Plasmodium* species (*P*. *falciparum*, *P*. *vivax*, *P*. *malariae*, *P*. *ovale*, and *P*. *knowlesi*) which puts more than 40% of the world’s population at risk [1]. Malaria also represents a risk for military operational readiness having caused greater loss of manpower in tropical regions than combat-related injuries [2].

Currently, malaria treatment in all endemic regions, regardless of their transmission intensity, is prescribed after only cases are confirmed with a diagnostic test that is either an RDT or light microscopy. However, both methods suffer from several drawbacks that limit their use. Light microscopy requires continuous power, highly skilled personnel, a long processing time and its sensitivity is affected by low parasitemia [3]. In the case of RDTs, their sensitivity is also limited in low parasitemia infections and their universal validity cannot be assured due to a high rate of false-negative results due to HRP2 and HRP3 deletions in *P*. *falciparum* [4–6].

Molecular approaches such as PCR and loop mediated isothermal amplification (LAMP) have been developed for highly sensitive and specific detection [7–9]. However, these methods can be relatively expensive and require laboratory capacities and trained personnel that are not readily available in endemic regions nor field conditions [10].

LAMP based methods have been widely explored and many variants have been tested showing good results in field and lab condition settings [11–13]. The optimal performance of LAMP is attributed to the use of the Bst polymerase which is considered more robust than conventional Taq polymerase and the use of 4–6 specific primers [14].

The photo- induced electron transfer (PET-PCR) is a real time assay that has been developed as a tool for malaria surveillance in endemic settings [15, 16]. This methodology has advantages over conventional nested-PCR and other Real- time platforms due the use of self-quenching primers, short processing time and the ease to perform the reaction [7]. The PET-PCR method can be used as a multiplexed platform allowing reducing time and cost [17].

However, there is still a need for high quality, point-of-care, cost-effective malaria diagnostic tools to enable detection of cases in the field in order to have prompt malaria detection that will not only improve patient health but will help to prevent transmission and reduce disease severity [1, 10].

In order to address this gap, we evaluated a new rapid, point-of-care, battery-operated device that detects hemozoin particles in the blood of infected patients named Gazelle. Hemozoin crystals are formed by the polymerization of dimers ferriprotoporphyrin IX that is a byproduct from hemoglobin digestion generated by all malaria species [18]. Previously attempts to use hemozoin as a biomarker for malaria diagnostics have been conducted [19–22], many of them have been unsuccessful and not applicable to field settings [18, 23].

However, there is promising evidence that magneto-optical detection can potentially allow sensitive identification of malaria. A previous in-vitro study on *P*. *falciparum* showed that this technology can potentially detect as low as 40 parasites per microliter of blood [24]. Furthermore, detection can occur since the early stages of infection and is maintained across different stages of the parasite lifecycle [25]. Furthermore, there is increasing clinical evidence that show that magneto-optical detection can be useful in *P*. *vivax* predominant elimination settings[26].

A previous study using Gazelle showed promising results for *P*. *falciparum* detection in a high-transmission setting in India [27]. Based on that study, we aimed to assess the Gazelle’s diagnostic performance in a low-transmission site with *P*. *vivax* predominance in the Peruvian Amazon Basin.

## Methods

### Study sites

**Fig 1:**
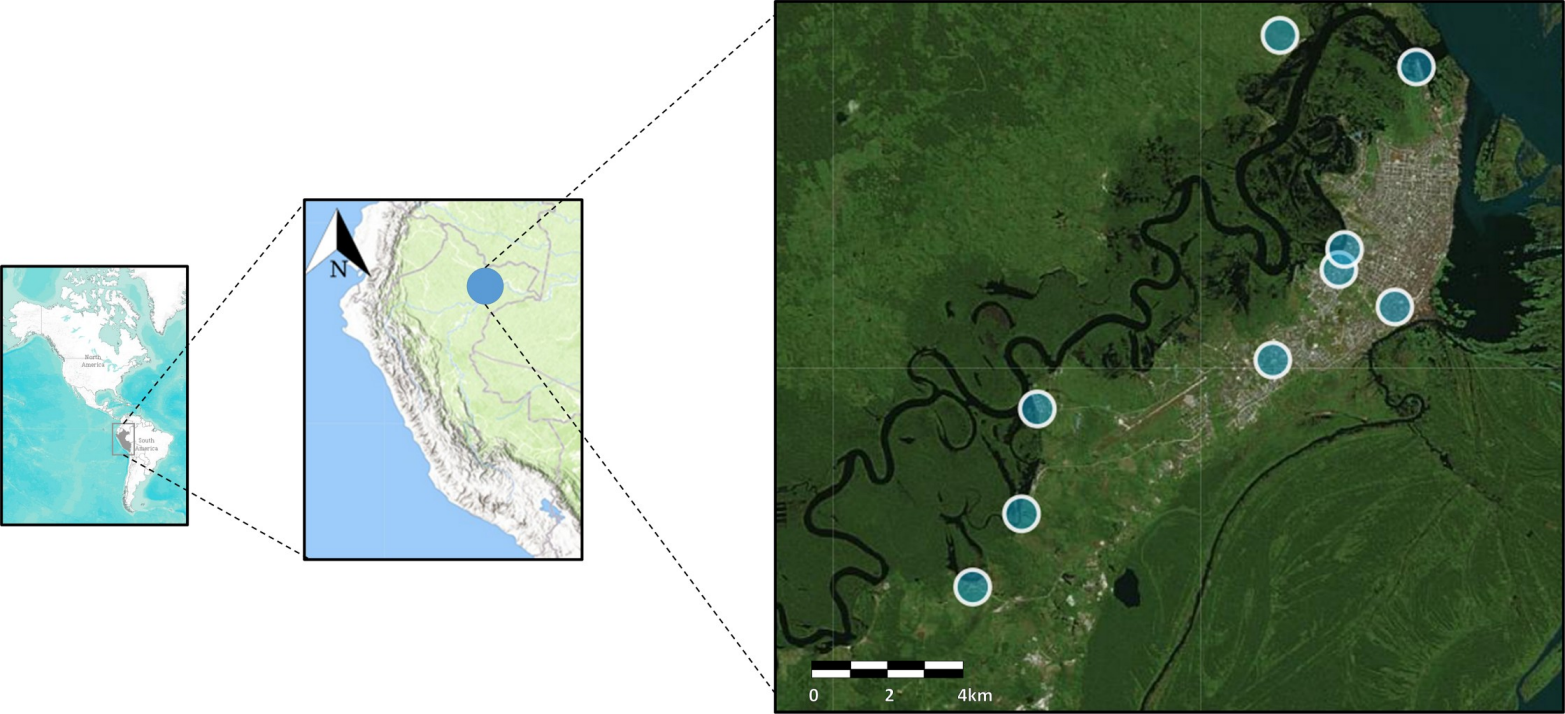
Study sites (blue circles) located in the city of Iquitos and surrounding communities. Map created using open data from openstreetmap.org: OpenStreetMap contributors.

Samples were collected from a passive surveillance study that enrolled febrile participants between 2019 and 2020 at nine health facilities located in the city of Iquitos and surrounding communities. Iquitos is the capital of the Loreto region which is located in the northwest side of the Peruvian Amazon basin (**Fig 1**). This region accounts for 28.7% of Peru’s territory with an incidence of malaria of 1.8% in 2019 (21,973 malaria cases reported in 2019) which represents more than 90% of all malaria cases reported by Peru during that year.

Malaria cases in Loreto are distributed in rural communities with little access to health care. The majority of cases are caused by *P*. *vivax* in a ratio of 4 to 1 with *P*. *falciparum*. Malaria diagnosis is primarily based on microscopy per the Peruvian national guidelines [28].

### Ethical considerations

The study protocol was approved by the Institutional Review Board of the U.S Naval Medical Research Unit 6 (NAMRU-6) in compliance with all applicable federal regulations governing the protection of human subjects (protocol NMRCD.2007.0004). Adult participants provided written consent and minors provided written assent and parental/guardian consent.

### Sample size

The sample size was estimated assuming a sensitivity of at least 70% for Gazelle with 10% relative precision at 95% confidence and 80% power. This resulted in a minimum sample size of 165 subjects.

### Sampling and malaria microscopy

Trained phlebotomists collected four mL of whole blood by venipuncture from each participant into sterile EDTA treated vacutainer tubes. Samples were transported under cold chain within two hours of collection to NAMRU-6 facilities in Iquitos. Two thin and thick blood smears were prepared for each participant, stained with 10% Giemsa and read by two microscopists at the NAMRU-6 laboratory in Iquitos. Results were provided after reading 200 oil-immersion fields and positive slides were quantified using 6,000 WBC/μl of blood as reference. Collected blood was used for RDTs and Gazelle testing in Iquitos and an aliquot was shipped to the NAMRU-6 laboratory in Lima for DNA extraction and molecular assays.

### RDT and Gazelle device testing

Two RDTs were used to diagnose malaria infection at the NAMRU-6 Iquitos laboratory by one laboratory technician: i) SD BIOLINE (05FK80-40-0) and ii) BinaxNOW (665–025). These two RDTs are based on the detection of the histidine-rich protein II (HRP-II) antigen of *P*. *falciparum* and lactate dehydrogenase (SD Bioline) or aldolase (BinaxNow) of *Plasmodium* species in human whole blood.

**Fig 2:**
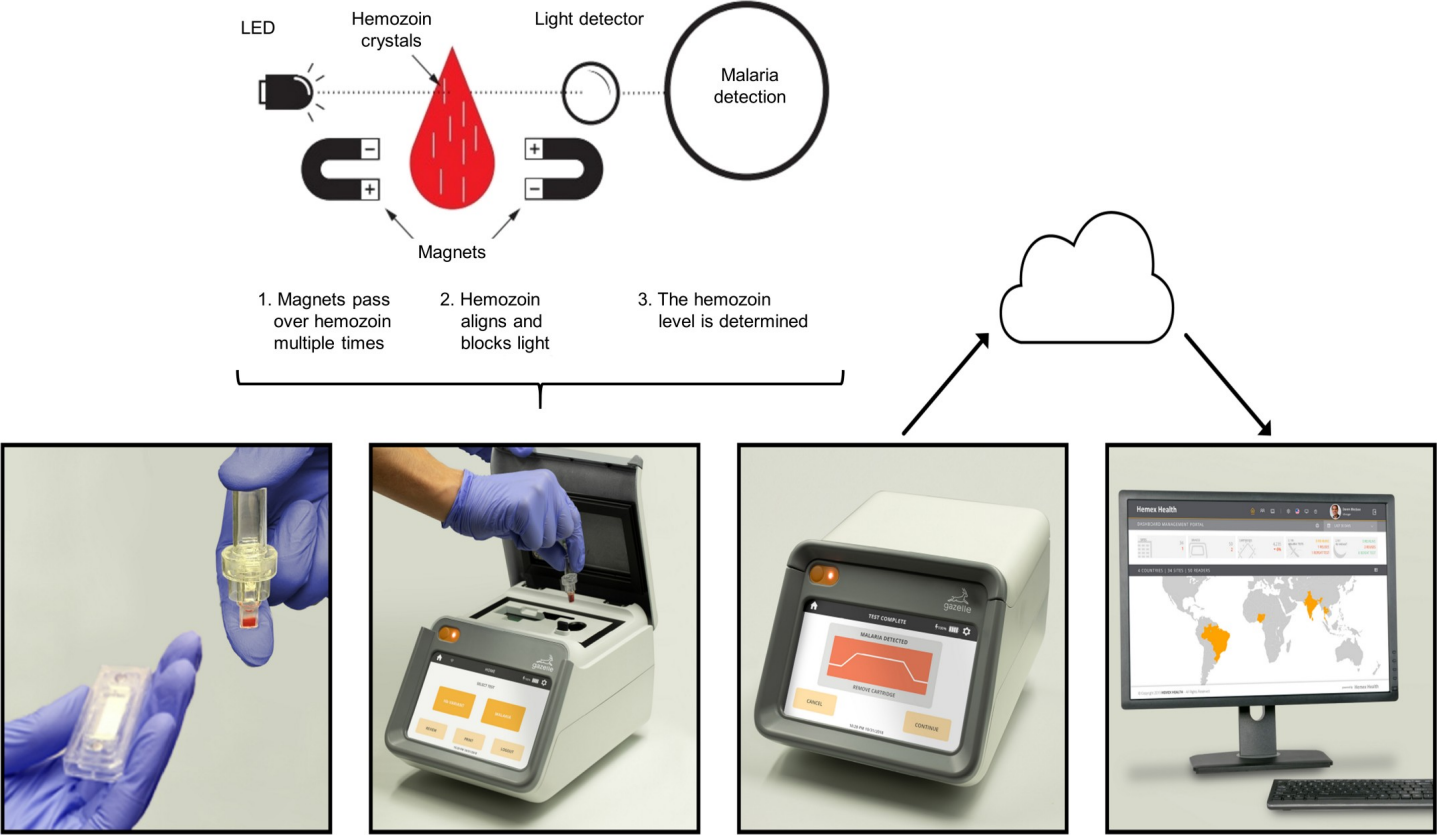
Gazelle device testing procedure. 15 μL of whole blood are put on a cartridge with 80 μL of whole blood Gazelle buffer. The sample is placed on the Gazelle device which lyse the sample by sonication and then pass magnets multiple times through the sample. Malaria detection is assessed by comparing the amount of light that traverse a sample with and without a magnetic field.

The Gazelle device testing was performed in the Iquitos laboratory by the same laboratory technician with fresh samples that were processed within four hours from collection. The device consists of a table-top reader and single-use disposable two-chamber cartridges (**Fig 2**). A pipette was used to drop 15 μL of whole blood into the lower chamber of the cartridge along with 80 μL of Gazelle buffer (2 % Triton in water). Then the upper chamber was placed, and the sample was sonicated through the wall of the cartridge, using a sonicator incorporated in the Gazelle.

The sonication step lyses red blood cells and releases hemozoin in positive samples. Then, internal magnets are passed multiple times over the sample and the resulting magnetic fields align the hemozoin crystals when present. This configuration is able to block the transmission of light through the sample. Finally, an internal LED emits light through the sample and the amount of light that traverses the sample is measured with and without the magnetic field (**Fig 2)**. The amount of light blocked is proportional to the quantity of hemozoin in the sample. The presence of any amount of hemozoin indicates malaria infection.

The result is displayed on the reader within one minute. The test theoretically detects all malaria species as hemozoin is produced by all of them, however it cannot differentiate between species. The device was calibrated with positive and negative controls every week or when switching the cartridges’ lot number. For the purpose of this study, Gazelle results were not displayed to keep the NAMRU-6 laboratory technicians blinded.

### Nested PCR

Nested PCR was conducted by two laboratory technicians in NAMRU-6 Lima. First, DNA was isolated from 200 μL of whole blood using the DNeasy Blood & Tissue kit (Qiagen, Germantown, MD) and eluted in 70 μL of elution buffer and used for 18S rRNA-nested PCR [29]. The first reaction amplifies the 18S rRNA subunit ribosomal (ssrRNA) gene and the second reaction targets specific regions for each plasmodium species (*P*. *falciparum* and *P*. *vivax)*. For the first reaction 5 μl of DNA template was used, while 5 μl of the amplified product were used in the second reaction. Both reactions were carried out in a 50 μl volume containing 1X buffer, 2mM MgCL2, 125μM dNTP’s, 250nM of each primer, and 1 unit of Taq Polymerase (Invitrogen, Waltham, MA). Results were visualized in a 2% agarose gel stained with GelRed®. Technicians were blinded to microscopy, RDT’s and Gazelle results.

### Photo-induced electron transfer (PET-PCR)

All samples were tested in duplicates using a *Plasmodium* genus PET-PCR in order to quantify parasite density [17, 30]. All genus positive samples were further tested by singleplex reactions to detect *P*. *falciparum* and *P*. *vivax*.

The genus reaction was carried out in a 20 μl volume that contained 5 μl of purified DNA, 2X TaqMan Environmental buffer 2.0 (Applied BioSystems, Foster City, CA), and 250nM of genus forward and FAM-reverse primer. The singleplex species-specific reactions contained the same mix but with a concentration of 125nM of the HEX-labelled species-specific primer.

PET-PCR reactions were run on an Mx3005P qPCR system (Agilent technologies, Santa Clara, CA) and results were visualized in the MXpro qPCR software. Thermal cycling conditions for the genus and species-specific PET-PCR consisted of an initial denaturation at 95°C for 10 minutes and 45 cycles of denaturation at 95°C for 10 sec followed by annealing at 60°C for 40 sec. A threshold cycle (Ct) below 41 was used to separate positive and negative samples. Technicians were blinded to microscopy, RDT’s and Gazelle results.

### Turn-around time and cost analysis

We estimated turn-around time and cost per sample for expert microscopy, Nested PCR, SD Bioline, BinaxNOW and Gazelle. The cost estimate included sample preparation for microscopy; DNA extraction, amplification reaction and results visualization for PCR; RDTs cost per sample and cartridge and buffer costs for Gazelle. Labor nor equipment cost were included in the analysis.

### Statistical methods

Sensitivity and specificity with 95% confidence intervals (95% CI) were calculated for microscopy, RDTs and Gazelle using 18S rRNA-nested PCR as reference test. The McNemar test was employed to detect statistical differences between microscopy, RDTs and Gazelle. The Kappa coefficient was used to estimate agreement between these diagnostic tests while the Mann-Whitney U test was used to assess differences in parasitemia between the Gazelle’s true positives and false negatives. These analyses were carried out in the “stats” package implemented in R [31]. Microscopy and PET-PCR quantification data were used to estimate the limit of detection (LoD) of Gazelle on clinical samples using bootstrapped cutpoints implemented in the “cutpointr” package in R.

## Results

### Enrolment and population characteristics

During the period of the study, 217 participants were enrolled and screened for malaria. A total of 32 participants were excluded from the final analysis because of inadequate blood sample to perform all four tests. Out of these 32 samples, microscopy identified 11 *P*. *vivax*, 3 *P*. *falciparum* and 18 negatives; BinaxNow resulted in 10 *non-P*. *falciparum* positives and 22 negatives whereas SDBioline resulted in 10 *P*. *vivax* and 22 negatives.

Out of the 185 remaining specimens, the malaria positivity rate by microscopy was 44.9% (83/185), by SD BIOLINE 38.9% (72/185), by BinaxNOW 35.6% (66/185), by Nested PCR 48.6% (90/185) and by Gazelle 43.8% (81/185) (**S1 Table**).

A total of four *P*. *falciparum* and one mixed *P*. *vivax/P*. *falciparum* cases were detected by Nested-PCR. All subsequent analysis were conducted with the remaining 180 specimens (85 *P*. *vivax* and 95 negatives as determined by Nested-PCR).

The median age was 33 years (range 5–79) and 60.5% were male. Participants had a median parasite density of 606 parasites/μL (12–61 773 parasites/μL) and recent history of antimalarial treatment was reported in 6 participants (**Table 1**).

**Table 1:** Characteristics of participants enrolled in the study.

Variable	Values
Study participants (N)	180
Median age (years)	33
Age range (years)	5–79
Sex (% males)	60.5%
Median parasite density in positive specimens (parasites per μL)	606
Parasitemia range in positive specimens (parasites per μL)	12–61 773
Malaria diagnosis in the previous month (N)	5
Antimalarial treatment in the previous month (N)	6

### Diagnostic performance of Gazelle and other diagnostic tests

**Fig 3:**
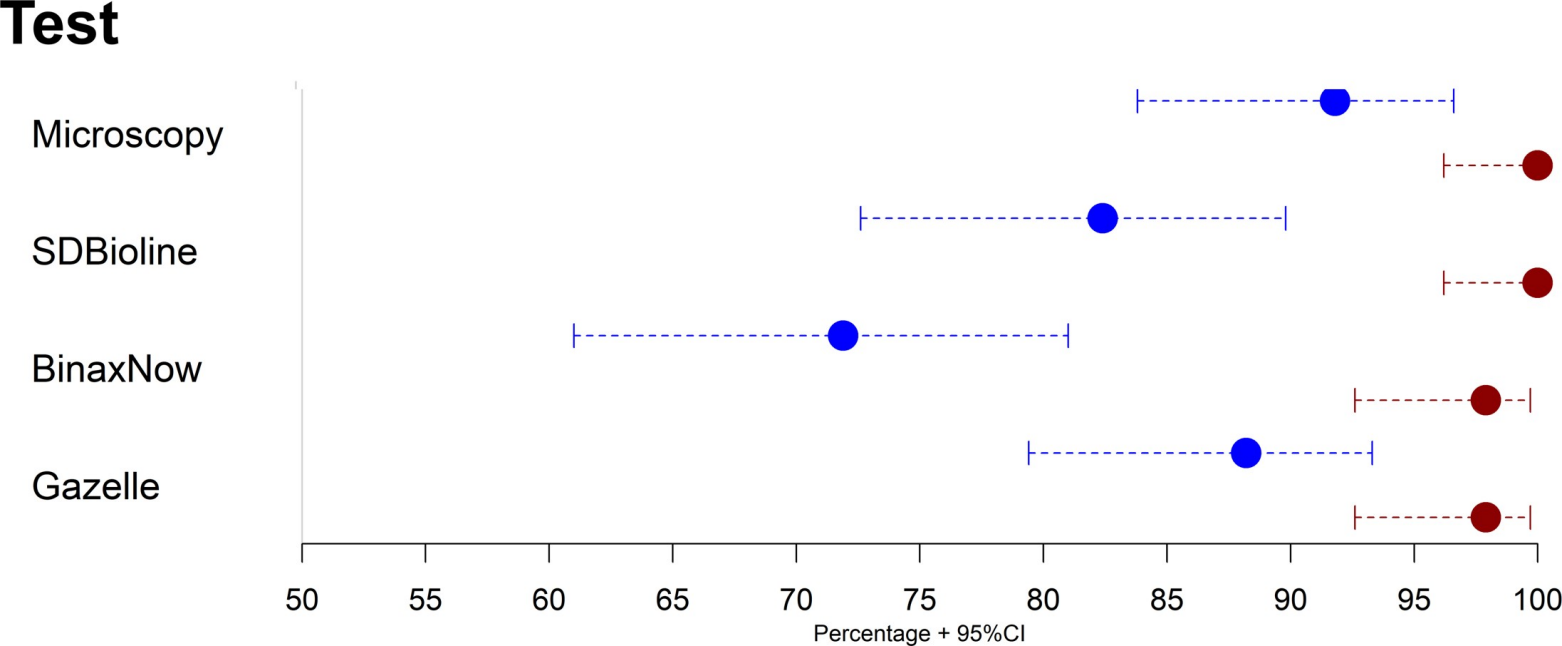
Sensitivity (blue dots), specificity (red dots) and 95% confidence intervals for detection of malaria by microscopy, SD Bioline, BinaxNOW and Gazelle in *P*. *vivax* using 18S rRNA-nested PCR as reference.

Using 18S rRNA-nested PCR as reference, microscopy had a sensitivity for *P*. *vivax* of 91.8% (95%CI: 83.8%– 96.6%) and specificity of 100% (95%CI: 96.2%– 100%). SD BIOLINE had a sensitivity of 82.4% (95%CI: 72.6%– 89.8%) and specificity of 100% (95%CI: 96.2%– 100%) whereas BinaxNOW had a sensitivity of 71.8% (95%CI: 61%– 81%) and specificity of 97.9% (95%CI: 92.6%– 99.7%). Gazelle had a sensitivity of 88.2% (95%CI: 79.4%– 94.2%) and specificity of 97.9% (95%CI: 92.6%– 99.7%) (**Fig 3**).

Using a cutoff point of 200 parasites/μl as in the WHO RDTs quality assurance program [32], the sensitivity of Gazelle for samples with more than 200 parasites/μl was 98.67% (95%CI: 92.79% to 99.97%) whereas the sensitivity for samples under 200 parasites/uL was 12.5% (95%CI: 0.32% to 52.65%).

**Fig 4:**
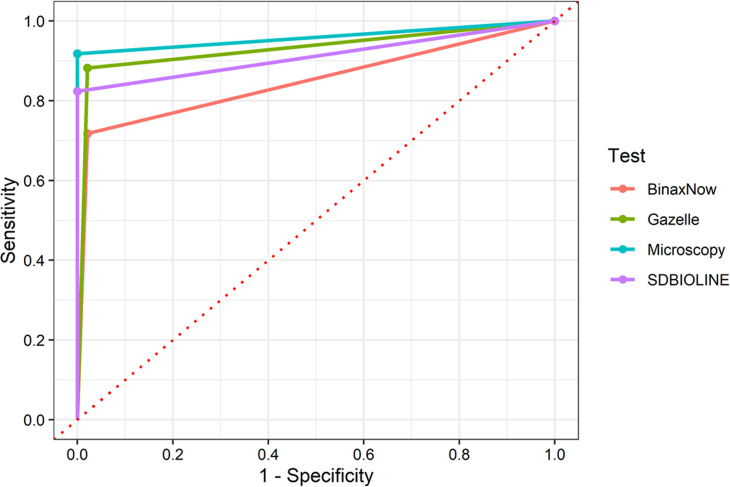
ROC analysis. The figure shows the ROC curves for BinaxNow, SD BIOLINE, microscopy and Gazelle compared with Nested PCR as reference test. Gazelle presented an AUC of 0.93 which is comparable to microscopy with an AUC of 0.96 and higher than BinaxNow (AUC = 0.85) and SD BIOLINE (AUC = 0.91).

Cohen’s kappa showed that Gazelle presented a kappa of 0.94 (95%CI: 0.89–0.99) compared with microscopy and a kappa of 0.87 (95%CI: 0.79–0.94) compared with nested PCR. The area under the ROC curve was 0.93 for Gazelle, 0.96 for microscopy, 0.91 for SD Bioline and 0.85 for BinaxNOW (**Fig 4**). The McNemar test did not show statistical differences between the results of Gazelle compared to microscopy (p = 0.983). However, statistical significant differences were found between Gazelle against both SD Bioline and BinaxNOW (p = 0.016 and p = 0.002, respectively).

### Limit of detection of Gazelle in P. vivax infections

Using 18S rRNA-nested PCR as reference, a total of 10 false negatives (FN) were found for Gazelle. Out of those, all were FN by both RDTs and 7 by microscopy. The average parasitemia in the 3 microscopy positive, but Gazelle FN specimens was 131 p/μl and the average parasitemia for submicroscopic, Gazelle FN was 1.4 p/ul using PET–PCR to quantify the parasitemia. Gazelle had 2 false positives (FP) results using 18S rRNA-nested PCR as reference. However, one FP turned out positive by PET-PCR with a parasitemia of 0.63 p/μl.

**Fig 5:**
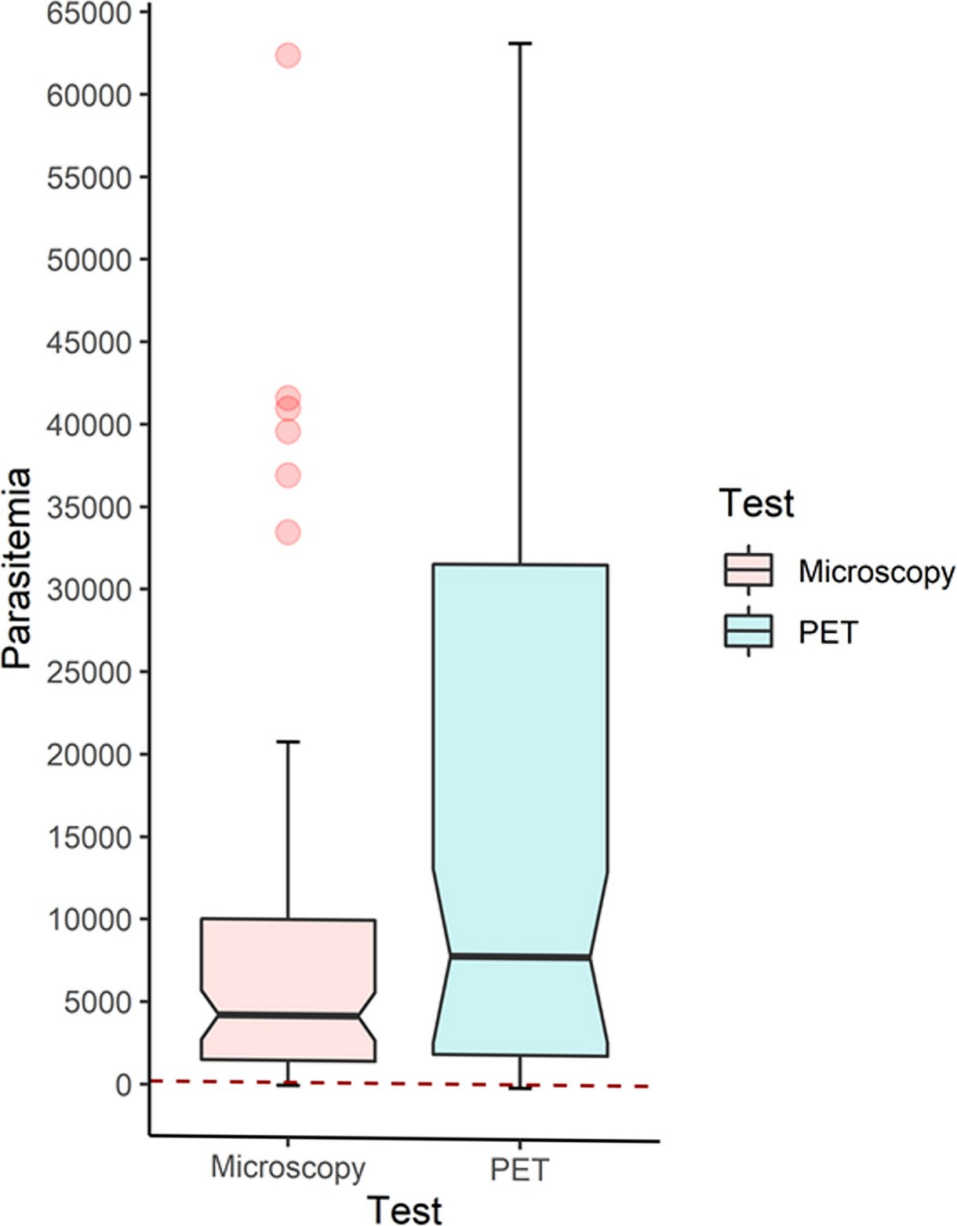
Parasite density distribution. The boxplot shows the distribution of parasite densities estimated by microscopy and PET-PCR along the median and lower and upper quartiles. The optimal parasitemia limit for a Gazelle positive result is shown as a dashed red line.

The Mann-Whitney U test showed significant differences in the median parasitemia of Gazelle true positives versus Gazelle FN (p<0.00001). In this regard, the optimal parasitemia limit for a Gazelle positive result was estimated between 207 and 323 parasites/μl using microscopy and PET-PCR quantification data, respectively (**Fig 5**).

The magneto-optical method used in this study is a quantitative technique as it detects hemozoin levels in the sample. In this regard, our results show a positive correlation between parasite density and the hemozoin signal detected by Gazelle (r = 0.83) (**S1 Fig**).

### Turnaround time and cost analysis

Our results show that Gazelle had a shorter turnaround time than SD Bioline and BinaxNOW with an estimated time of five minutes per test including sample preparation and reading (**Table 2**). Gazelle cost per test was estimated at one US dollar per test which is lower than BinaxNOW and similar to microscopy and SD Bioline (**Table 2**).

**Table 2:** Turnaround time and cost analysis. Estimated cost covers reagents and supplies for DNA extraction and slide preparation. No equipment no labor costs are included.

Procedures	Cost (USD) per sample	Turnaround time (minutes)	Hands-on work (Minutes)
**Microscopy**	$0.94	120	40
**Nested-PCR**	$ 9.05	540	80
**SD Bioline**	$ 0.60	20	5
**BinaxNOW**	$ 42.85	20	5
**Gazelle**	$ 1.00	5	3

## Discussion

Current guidelines indicate that malaria treatment should be provided only to cases with a positive result by a diagnostic test, which is either an RDT or light microscopy [10]. Furthermore, malaria diagnostics are not only key to direct treatment but are also critical for surveillance and identify transmission reservoirs in endemic settings [10]. However, RDT and light microscopy present several limitations that impairs their use in the field. Therefore, novel field-deployable approaches are needed for point-of-care proactive case detection in endemic settings.

In this study, we evaluated a novel rapid point-of-care diagnostic device (Gazelle) and assessed its performance against microscopy and two RDTs (SD Bioline and BinaxNOW) using Nested PCR as the gold standard. The results of the current study showed that Gazelle’s sensitivity and specificity were similar to microscopy and better than RDTs which are the primary diagnostic methods used in health centers in Peru and other endemic regions.

Our results are in line with a recent study on the performance of Gazelle in a high-transmission region in India with predominance of *P*. *falciparum* [27].The previous study showed that Gazelle presented a sensitivity and specificity of 82% and 98%, respectively; which are close to the 88.2% and 97.9% values of sensitivity and specificity from our study. These results suggest that the diagnostic performance of Gazelle is similar for *P*. *falciparum* and *P*. *vivax* cases.

This is an important finding since species predominance varies between endemic regions. Furthermore, shifts in predominant species can occur within the same region as a result of malaria control activities, which tend to be more effective against *P*. *falciparum* than *P*. *vivax* or introduction of new strains as previously shown [33–36].

Gazelle’s turnaround time is fast (5 minutes) compared to microscopy (2 hours), RDTs (20 minutes) and PCR (9 hours) and the cost per test is comparable to microscopy and RDTs. Furthermore, Gazelle’s device cost is approximately $700 which is comparable to the cost of a basic light microscope and the device can process more than 20,000 tests during its estimated lifecycle.

In this regard, our data suggests that Gazelle could complement microscopy or could be used as an alternative for malaria diagnosis in remote or resource-limited settings. This potential for point of care diagnosis is further supported by the minimal training required for Gazelle operation and the lack of observer-dependent variability as in microscopy.

Using the WHO RDTs quality assurance program as a reference, the sensitivity of Gazelle for samples with high parasite density (>200 parasites/μl) was 97.47% (95%CI: 91.15% to 99.69%). This is a relevant finding as a density of 200 parasites/μl is well below the mean parasite density reported in many endemic settings including our sites in Peru [32]. Furthermore, the false positive rate of Gazelle was estimated in 2.1% which is below current WHO criteria for RDTs (<10%) [32].

The limited sample size (n = 10) for extremely low parasitemia infections (<200 parasites/μl) did not allow us to calculate a robust sensitivity estimate for Gazelle. However, given that the magneto-optical method used here is a quantitative technique, it could be possible to increase the sensitivity for low parasitemia infections by modifying the input sample volume.

It is important to highlight that the current Gazelle prototype does not have the capacity for species identification. This an important drawback in regions where multiple species circulate and where treatment schemes differ since incorrect case management can lead to poor response to treatment with worsening disease, relapse or recrudescence [37]. In this regard, the capacity for species differentiation is currently being incorporated into the next device prototype that will be validated in the near future.

Gazelle constitutes an important addition to the malaria diagnostic toolkit that is suitable for point-of-care malaria detection. Our results show the potential of Gazelle as a cost-effective malaria diagnosis device that can be used in resource-limited endemic settings. Knowledge gained from this study will inform further advances in translating the technology into an affordable, rugged, and operational ready device that can be utilized in all malaria-endemic regions.

## Supporting information

S1 Fig Scatterplot of parasitemia versus quantitative magneto-optical data. The figure shows microscopy determined parasitemia versus Gazelle’s hemozoin quantification. A positive correlation was found between both measures (r = 0.83).
(TIF)

S1 Table Malaria detection results. The table shows the results for each of the methods used in this study for each of the samples as well as basic data from each of the participants including age, sex and date of sample collection.
(XLSX)
