# Supplementary figures and images for: Field validation of a magneto-optical detection device (Gazelle) for portable point-of-care Plasmodium vivax diagnosis
Source: PLoS One. 2021 Jun 22;16(6):e0253232. doi: 10.1371/journal.pone.0253232 (PMC8219132; doi:10.1371/journal.pone.0253232)

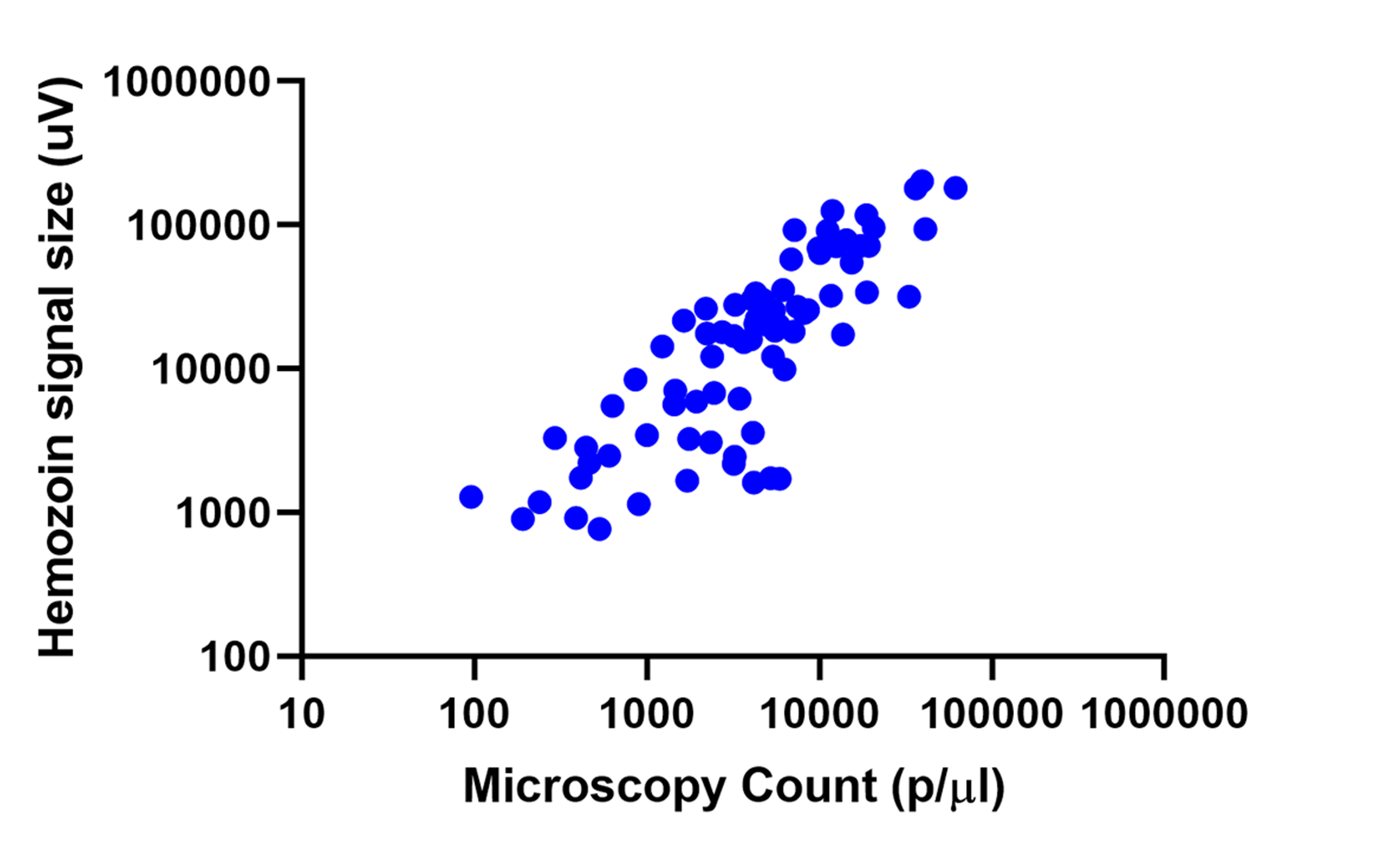

Supplement: S1 Fig — The figure shows microscopy determined parasitemia versus Gazelle’s hemozoin quantification. A positive correlation was found between both measures (r = 0.83). (TIF) [file pone.0253232.s001.tif]
